# Supplementary material for: The genome sequence of Geoffroy’s Bat, Myotis emarginatus (Geoffroy, 1806) (Chiroptera: Vespertilionidae)
Source: Wellcome Open Res. 2026 May 2;11:258. [Version 1] doi: 10.12688/wellcomeopenres.26492.1 (PMC13227098; doi:10.12688/wellcomeopenres.26492.1)
Supplement: Peer review history

# Reviewer report: Reviewer response for version 1

**DOI:** 10.21956/wellcomeopenres.29173.r154838

**Published:** 2026-06-05

**Authors:** Michael R Buchalski, Samantha Capel

**Affiliations:** 1 Wildlife Health Laboratory, California Department of Fish and Wildlife, Sacramento, CA, USA

This article presents a reference genome assembly for *Myotis emarginatus*, detailing the sequencing and assembly methodology and resultant assembly quality. All key methodology and results are present, but there are a few details missing:

 1. Tissue type from which RNA was extracted is not specified in-text within the "RNA library preparation and sequencing" Methods subsection

 2. Scaffold and contig L50 scores should be included in Table 2

 3. Why wasn't the RNA seq data used to annotate the genome? You may want to specify as readers will be curious.

 4. You claim to have a chromosome-level assembly but make no reference to known Myotis karyotypes and how they relate to your final assembly structure

Are sufficient details of methods and materials provided to allow replication by others?

Yes

Is the rationale for creating the dataset(s) clearly described?

Yes

Are the datasets clearly presented in a useable and accessible format?

Yes

Are the protocols appropriate and is the work technically sound?

Yes

Reviewer Expertise:

Population genomics, conservation genomics, bat genomics

We confirm that we have read this submission and believe that we have an appropriate level of expertise to confirm that it is of an acceptable scientific standard, however we have significant reservations, as outlined above.

# Reviewer report: Reviewer response for version 1

**DOI:** 10.21956/wellcomeopenres.29173.r154837

**Published:** 2026-06-01

**Authors:** Keping Sun

**Affiliations:** 1 Northeast Normal University, Changchun, Jilin, China

In the Introduction section, please add relevant literature to support the description of the diet of this species.

 In the sample acquisition section, please indicate which organs were collected.

 For the species identification, the authors state: “The specimen was identified by Manuel Ruedi based on morphological and mitochondrial DNA characters [GenBank OQ706656 and OQ885399; Ruedi et al. (2023)].” Please specify which mitochondrial gene(s) were used, or whether the entire mitochondrial genome was sequenced.

 For the DNA extraction, please indicate which organs were used for DNA extraction. For the RNA extraction, please clarify why RNA was extracted from spleen tissue.

 References with missing page numbers:

 Danecek P, Bonfield JK, Liddle J, *et al.* : Twelve years of SAMtools and BCFtools. *GigaScience.* 2021; **10** (2).

 Howe K, Chow W, Collins J, *et al.* : Significantly improving the quality of genome assemblies through curation. *GigaScience.* 2021; **10** (1)

 Merkel D: Docker: Lightweight Linux containers for consistent development and deployment. *Linux J.* 2014; **2014** (239): 10.5555/2600239.2600241.

 Rhie A, Walenz BP, Koren S, *et al.* : Merqury: Reference-free quality, completeness, and phasing assessment for genome assemblies. *Genome Biol.* 2020; **21** (1).

 Zhou C, McCarthy SA, Durbin R: YaHS: Yet another Hi-C scaffolding tool. *Bioinformatics.* 2023; **39** (1).

Are sufficient details of methods and materials provided to allow replication by others?

Yes

Is the rationale for creating the dataset(s) clearly described?

Yes

Are the datasets clearly presented in a useable and accessible format?

Yes

Are the protocols appropriate and is the work technically sound?

Yes

Reviewer Expertise:

animal molecular ecology

I confirm that I have read this submission and believe that I have an appropriate level of expertise to confirm that it is of an acceptable scientific standard.

# Reviewer report: Reviewer response for version 1

**DOI:** 10.21956/wellcomeopenres.29173.r154832

**Published:** 2026-05-27

**Authors:** Heliana Dundarova

**Affiliations:** 1 Institute of Biodiversity and Ecosystem Research, Bulgarian Academy of Sciences, Sofia, Bulgaria

This data note presents a chromosome-level genome assembly of *Myotis emarginatus* generated within the framework of the Darwin Tree of Life Project. Overall, this is a very solid and well-prepared genome note that provides an important genomic resource for future studies on bat evolution, comparative genomics, ecology, and conservation.

 It is clearly written, the methodology is appropriate and described in sufficient detail, and the assembly statistics demonstrate a high-quality genome. The use of PacBio HiFi sequencing combined with Hi-C scaffolding and manual curation resulted in an assembly with excellent continuity and completeness. 

 The manuscript follows current standards for genome resource publications and will be useful to a broad community of researchers working on bats and vertebrate genomics.

 The background section is concise but informative, especially regarding the ecology and phylogenetic position of *M. emarginatus*. The discussion of the species as the only European representative of the African clade of *Myotis* adds evolutionary relevance to the study.

Are sufficient details of methods and materials provided to allow replication by others?

Yes

Is the rationale for creating the dataset(s) clearly described?

Yes

Are the datasets clearly presented in a useable and accessible format?

Yes

Are the protocols appropriate and is the work technically sound?

Yes

Reviewer Expertise:

Ecology, evolution, and conservation of Eurasian bats, with particular focus on the role of bats as hosts and vectors of RNA viruses.

I confirm that I have read this submission and believe that I have an appropriate level of expertise to confirm that it is of an acceptable scientific standard.
